# The Premature Ejaculation Diagnostic Tool (PEDT): Translation, Psychometric Validation, and Cross-Cultural Adaptation of an Urdu Version (PEDT-U)

**DOI:** 10.7759/cureus.113107

**Published:** 2026-07-21

**Authors:** Khaleeq Ur Rehman, Usama Muhammad Kathia, Muhammad Waqas, Sharoon Hanook, Noor Zohra, Muhammad Asif Mahmood, Khalid M Anjum

**Affiliations:** 1 Urology, Fatima Memorial Hospital (FMH) College of Medicine and Dentistry, Lahore, PAK; 2 Andrology and Infertility, Hameed Latif Hospital, Lahore Institute of Fertility and Endocrinology (LIFE), Lahore, PAK; 3 Urology, Services Hospital, Lahore, PAK; 4 Urology, Mayo Hospital, Lahore, PAK; 5 Biostatistics, Forman Christian College (A Chartered University), Lahore, PAK; 6 Psychiatry and Behavioral Sciences, Fatima Memorial Hospital (FMH) College of Medicine and Dentistry, Lahore, PAK; 7 Diabetology Out-Patient, Ittefaq Hospital, Lahore, PAK; 8 Biostatistics, Fatima Memorial Hospital (FMH) College of Medicine and Dentistry, Lahore, PAK

**Keywords:** intravaginal ejaculatory latency time (ielt), pedt, premature ejaculation (pe), psychometric validation, questionnaire, translation, urdu

## Abstract

Premature ejaculation (PE) is a common type of male sexual dysfunction. The Premature Ejaculation Diagnostic Tool (PEDT) was developed as a standardized tool to correctly diagnose PE and has been translated into many languages. However, a valid, culturally adapted Urdu version of the PEDT (PEDT-U) for northern Punjab does not exist. This study aimed to develop the Urdu version of the PEDT-U and evaluate its psychometric properties in a sample of Pakistani men with PE compared with healthy controls. PEDT-U was translated by two blinded translators and further refined using the forward-backward translation method. A total of 168 bilingual participants, 87 (51.79%) of them clinically diagnosed with PE (PE group) and 81 (48.21%) controls, completed both PEDT and PEDT-U questionnaires. Response time for PEDT-U was significantly less (p < 0.001). Receiver operating characteristic curve analysis showed that the area under the curve (AUC) for the PEDT-U was 0.987 (95% CI: 0.974-1.0), with a sensitivity of 0.736 and a specificity of 0.988. For the PEDT, the AUC was also 0.987 (95% CI: 0.976-0.999), with a sensitivity of 0.747 and a specificity of 0.990. Cronbach's alpha scores for PEDT (0.938) and PEDT-U (0.939) showed adequate internal consistency in both questionnaires. Cohen's kappa statistic showed good agreement in the responses of the participants; the median intravaginal ejaculatory latency time (IELT) for the 28 PE patients was 45 s, with a minimum IELT of 12 s and a maximum of 71 s. PEDT-U has shown a significant correlation between the original English and the translated PEDT-U. It has shown good internal consistency, test-retest reliability, degree of agreement, and discriminant validity relative to the original English version of the PEDT. It takes less time, and patients felt more comfortable with it. Hence, this tool is recommended for the diagnosis of PE among the Pakistani population.

## Introduction

Premature ejaculation (PE) is a common type of male sexual dysfunction. It is the most common sexual dysfunction, affecting 25-40% of males across different age groups, imposing psychological and physical burdens, and also causing relationship problems [1]. The patient may be a lifelong sufferer from this disorder or may have developed it later in life. Currently, assessment of patients with PE relies mainly on the use of validated questionnaires [2]. Various tools have been developed and validated, including the Chinese Index of PE [3], the Index of PE [4], the PE Diagnostic Tool [5], the Arabic Index of PE [6], and the PE Profile [7].

The Second International Society of Sexual Medicine (ISSM) Ad Hoc Committee in 2013 defined premature ejaculation (lifelong and acquired PE) as the inability to delay ejaculation beyond 1 min in lifelong PE and beyond 3 min in acquired PE with negative personal consequences [8,9].

The committee agreed that the published objective evidence on PE is limited only to the studies of men with PE engaging in vaginal intercourse, but there is insufficient information to objectively define problematic early ejaculation in the context of oral sex, anal sex, and same-gender sexual activity.

The Premature Ejaculation Diagnostic Tool (PEDT) has been translated into various languages, including Chinese, Turkish, Spanish, Colombian, and Malay [10-13]. A translated version of the PEDT into Urdu for use in Pakistan was developed by Bangash et al. [14]. However, although the Urdu version of the PEDT is suitable for diagnosing PE in populations living in southern Pakistan, dialectal differences in northern Pakistan necessitated the development of the PEDT-U for use in that population. The original English PEDT was translated into the Urdu version (PEDT-U) according to the American Association of Orthopaedic Surgeons (AAOS) Outcomes Committee's recommendations, as described below [15]. Permission to use the translated version of the PEDT has been obtained from the original copyright holder. This study aimed to translate and cross-culturally adapt the original English PEDT into Urdu and to establish the psychometric validity of this new diagnostic tool (PEDT-U).

## Materials and methods

The study was conducted after receiving approval from the Institutional Review Board (IRB) of the Fatima Memorial Hospital (FMH), College of Medicine and Dentistry, and each participant provided written informed consent to participate. This is a cross-sectional study in which clinically diagnosed cases of PE and appropriate controls, who had no PE but presented to the urology OPD with other urological complaints, were included. The original PEDT English questionnaire (PEDT) was translated into the PEDT Urdu (PEDT-U) version according to the American Association of Orthopaedic Surgeons (AAOS) Outcomes Committee's recommendations defined below [15].

Stage I: initial translation

The PEDT-U was translated by two independent translators who were blinded to the original version to produce a finalized version of the questionnaire. It was further refined using the forward-backward translation method. These translators were blind to the original version of the questionnaire.

Stage II: synthesis of the translations

After obtaining two translated versions from both translators, a finalized version of the translation was derived. After careful comparison of the translated versions of PEDT-U and PEDT, the discrepancies in PEDT-U were identified and addressed. For experiential equivalence, the items were simplified to clarify the wording. Similarly, a committee of experts reviewed the questionnaire for its conceptual equivalence and cultural equivalence with the culture of northern Pakistan.

Stage III: back translation

After finalizing the version, two separate translators who were totally blind to the original PEDT questionnaire reverse-translated PEDT-U into English PEDT. The reverse-translated English version of PEDT-U and the original English version of PEDT were compared with each other by the panel of experts.

Stage IV: expert committeet of premature ejaculation

The expert committee combined all versions of the questionnaire and developed a final version of PEDT-U for pretesting. For semantic equivalence, the version was reviewed for any grammatical errors or any similar wordings used in the questionnaire. For idiomatic equivalence, any words difficult to understand were replaced with simpler words by the group of experts. For experiential equivalence, the questionnaire items were replaced with similar items according to the culture of Pakistan. For the purpose of conceptual equivalence, the expert committee reviewed the translated version for any cultural difficulties in the questionnaire's language to ensure clarity.

Stage V: test of the pre-final version

In this step, the finalized version was pre-tested on 10 consenting males who visited the urology outpatient department with the complaints of premature ejaculation and were asked about any difficulties and suggestions for the questionnaire. These discrepancies were modified to bring clarity. Both the meaning of the items and responses were explored. This ensured that the translated version still retained its equivalence.

Stage VI: submission of documentation to the coordinating committee for appraisal of the adaptation process

In this step, the final testing of the questionnaire was done to ensure uniformity between both versions of the questionnaire.

Characteristics of the participants

After obtaining a finalized version of the questionnaire for the purpose of cross-cultural validation, 168 males were recruited. A total of 87 (51.79%) participants clinically diagnosed with PE were included in the PE group, whereas 81 (48.21%) participants were placed in the control group, who did not have PE but presented to the urology OPD with some other complaint.

Participants aged 18-60 years, in a stable married relationship for at least six months, clinically diagnosed with PE, and consenting to participate in the study were included. Those patients who had a clinical diagnosis of erectile dysfunction (ED) or were taking medications for sexual problems, like phosphodiesterase 5 inhibitors, and had any mental or medical condition like depression, cardiovascular disease, hypertension, and diabetes mellitus were excluded from the study.

Agreement between responses on both versions

The participants were requested to fill in the English version and then the Urdu version of the questionnaire to see the agreement between the two versions.

Clinical evaluation of responses

For the purpose of clinical evaluation, the participants were interviewed by the senior consultant andrologist/urologist, who was blinded to their responses in both the English and Urdu versions of the questionnaires. In addition to this, the intravaginal ejaculatory latency time (IELT) was taken from 28 individuals in the clinically diagnosed PE group. It was done to augment the validity of PEDT in the PE group.

Statistical analysis

The statistical analysis for the psychometric validation and cross-cultural adaptation of PEDT-U was performed using IBM SPSS version 18 (Armonk, NY: IBM Corp.). Wilcoxon signed-rank sum test was performed to observe discriminant validity among the responses obtained for PEDT and PEDT-U versions; Cohen's kappa was obtained to observe the degree of agreement between the PEDT and PEDT-U. Sensitivity and specificity of PEDT-U scores were evaluated using receiver operating characteristic (ROC) analysis. Internal consistency of the questionnaire was observed using Cronbach's alpha, which was considered a good level of internal consistency. Test-retest reliability was performed using Spearman's correlation coefficient. The analysis was carried out between the PEDT and PEDT-U in both the clinically diagnosed PE group and controls. Further analysis was conducted comparing the clinical diagnosis and the PEDT-U diagnosis; all statistical tests were conducted at a 5% significance level.

## Results

A total of 168 participants completed the PEDT and PEDT-U questionnaires; 87 (51.79%) clinically diagnosed with PE (PE group) and 81 (48.21%) controls were asked to respond to the PEDT questionnaires. Cronbach’s alpha scores for PEDT (0.938) and for PEDT-U (0.939) showed adequate internal consistency in both questionnaires. On average, participants took significantly less time to complete the PEDT-U (p < 0.001). The average PEDT scores obtained using PEDT and PEDT-U were the same for both questionnaires (p = 0.704) (Table 1).

**Table 1:** Comparison of clinical diagnosis vs. PEDT-U and PEDT. *Median (min-max). **Wilcoxon signed-rank test. PEDT: Premature Ejaculation Diagnostic Tool; PE: premature ejaculation

Variables	PEDT English version, n = 168			PEDT Urdu version, n = 168			p-Value**
	PE group 87 (51.79%)	Controls 81 (48.21%)	Total 168 (100%)	PE group 87 (51.79%)	Controls 81 (48.21%)	Total 168 (100%)	
PEDT score*	14 (3-20)	0 (0-10)	6 (0-20)	14 (3-20)	0 (0-13)	6.5 (0-20)	0.704
Time (s)*	300 (60-900)	240 (120-1200)	300 (60-1200)	240 (50-480)	120 (30-600)	180 (30-600)	<0.001
No PE, n (%)	14 (15.05)	79 (84.95)	93 (55.7)	15 (15.96)	79 (84.04)	94 (55.95)	-
Borderline PE, n (%)	8 (80.0)	2 (20.0)	10 (6.0)	8 (88.89)	1 (11.11)	9 (5.36)	
PE, n (%)	65 (100)	0 (0)	65 (38.3)	64 (98.46)	1 (1.54)	65 (38.69)	

The questionnaire consists of responses ranging from 0 to 4, with higher scores indicating a higher level of sexual function impairment. There were five questions; therefore, the maximum possible questionnaire score was 20, and the minimum possible score was 0. Based on the scores, the PE status was described as “PE” (score ≥ 11), “borderline PE” (score 9-10), and “no PE” (score ≤ 8).

Spearman's correlation coefficients (rs ≥ 0.977) indicated higher test-retest reliability and greater similarity among the items of PEDT and PEDT-U. Strong agreement was observed, with a Spearman's correlation coefficient of 0.995 (p < 0.001) between the total PEDT and PEDT-U scores (Table 2).

**Table 2:** Test-retest reliability using Spearman's correlation coefficient (rs) for each of the five items. PEDT: Premature Ejaculation Diagnostic Tool; PEDT-U: Premature Ejaculation Diagnostic Tool Urdu version

Question	1	2	3	4	5	Total score
PEDT vs. PEDT-U	0.994	0.983	0.977	0.987	0.983	0.995
P-value	<0.001	<0.001	<0.001	<0.001	<0.001	<0.001

**Figure 1:**
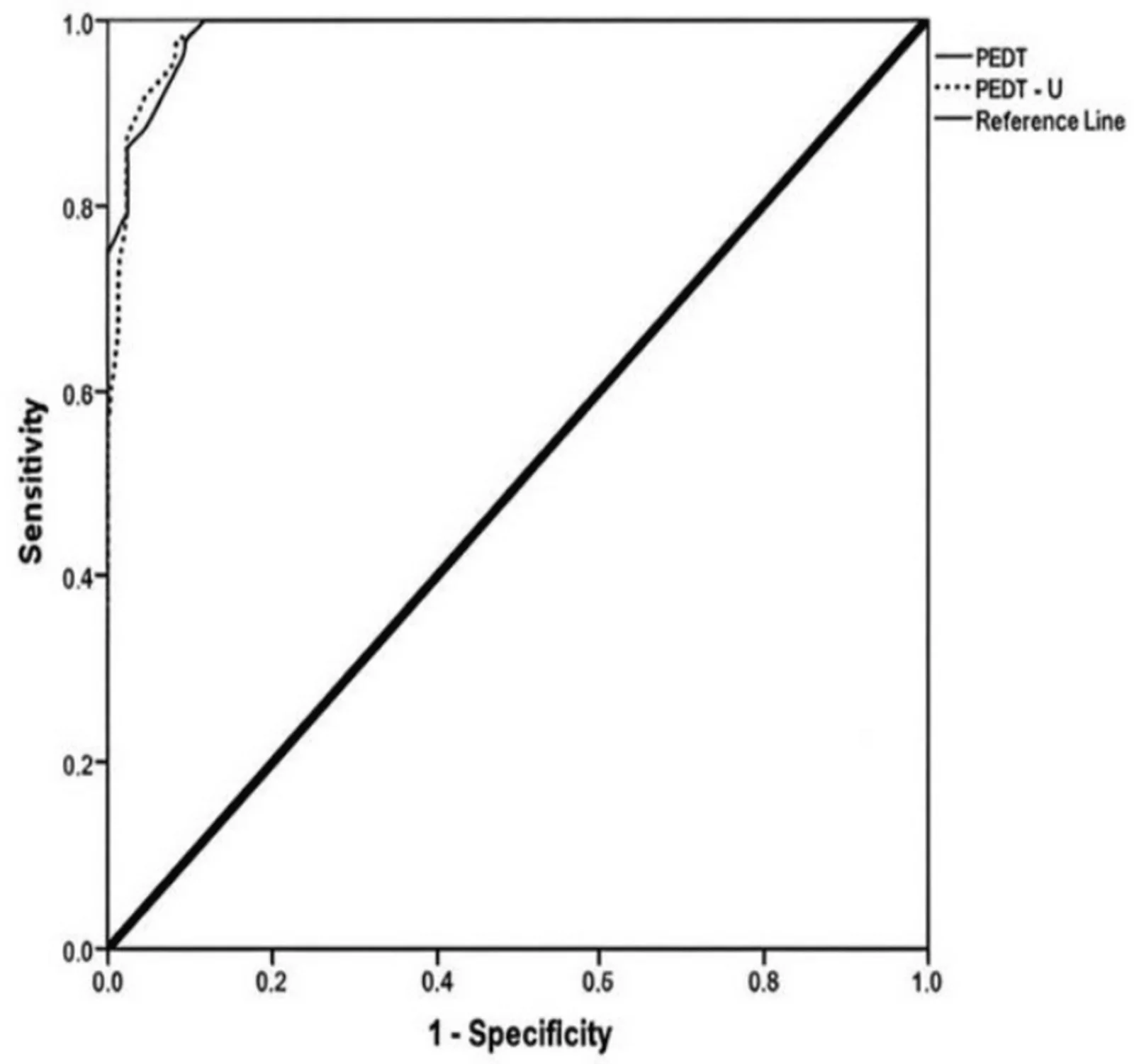
Receiver operating characteristic curve for PEDT and PEDT-U vs. clinical diagnosis. PEDT: Premature Ejaculation Diagnostic Tool; PEDT-U: Premature Ejaculation Diagnostic Tool Urdu version

Receiver operating characteristic (ROC) curve analysis comparing the PEDT-U with the clinical diagnosis, using a cutoff score of ≤10 to indicate no PE and ≥11 to indicate PE, yielded an area under the curve (AUC) of 0.987 (95% CI: 0.974-1.0), with a sensitivity of 0.736 and a specificity of 0.988. For the PEDT, the AUC was 0.987 (95% CI: 0.976-0.999), with a sensitivity of 0.747 and a specificity of 0.990 (Figure 1).

Intravaginal ejaculatory latency time (IELT) was taken from 28 individuals in the clinically diagnosed PE group. It was done to augment the validity of PEDT in the clinically diagnosed PE group; the median IELT for the 28 PE patients was 45 s, with a minimum IELT of 12 s and a maximum of 71 s.

The concordance among the responses from the PEDT and PEDT-U versions using Cohen's kappa statistic showed good agreement between the responses of the participants. To further examine whether there was any difference in the responses to the individual questions in the PEDT and PEDT-U, the results of the Wilcoxon signed-rank test indicated no significant differences in responses to any question between the PEDT and PEDT-U (Table 3).

**Table 3:** Concordance among PEDT and PEDT-U. *Wilcoxon signed-rank Z-statistic. **Wilcoxon signed-rank test. Q1_E: question number 1 in English; Q1_U: question number 1 in Urdu, Q2_E: question number 2 in English; Q2_U: question number 2 in Urdu; Q3_E: question number 3 in English; Q3_U: question number 3 in Urdu; Q4_E: question number 4 in English; Q4_U: question number 4 in Urdu; Q5_E: question number 5 in English; Q5_U: question number 5 in Urdu

Comparison	Cohen's kappa	p-Value	Z*	p-Value**
Q1_U vs. Q1_E	0.977	0	-0.816	0.414
Q2_U vs. Q2_E	0.929	0	-1.811	0.070
Q3_U vs. Q3_E	0.944	0	-1.0	0.317
Q4_U vs. Q4_E	0.950	0	-0.647	0.518
Q5_U vs. Q5_E	0.939	0	-0.877	0.380

## Discussion

Sexual dysfunction is a common issue. Primary healthcare professionals, including urologists and surgeons, need to be able to recognize, diagnose, and treat such conditions. Recognizing patients' needs is often challenging for clinicians and patients alike. Self-administered questionnaires are effective in breaking the silence between the patient and his physician. So, we have translated the PEDT questionnaire into Urdu, the national and local language of Pakistan, which is widely understood by the literate population in northern Pakistan.

Translation and adaptation of the original English questionnaires into Urdu for cross-cultural and psychometric equivalence give rise to difficulties, as suitable terms are not easily adaptable [16]. Discussing one's sexuality is considered a social taboo, and therefore it is often discussed secretly in local languages.

It should be noted that Urdu is spoken by almost all ethnic groups in the country and is taught as a subject in most schools. In northern Pakistan, people also speak other indigenous languages such as Punjabi, Pashto, Balti, Kohistani, Shina, and Kashmiri, depending on their ethnicity and geographic location. The advantage of this translation into Urdu is that it is understood by people of northern Pakistan, irrespective of their diversity. One of the limitations of this study was that all individuals recruited had a good command of both English and Urdu and were literate.

Validation of the Urdu version of PEDT

The translation of various questionnaires into different languages for widespread use is very common. Many such questionnaires have been translated previously and successfully tested for their validity and cross-cultural adaptation in local populations worldwide [8]. Previous studies have repeatedly shown that each PE dimension is substantially influenced by cultural factors and that men's perceptions of PE differ significantly across cultures. Therefore, cultural adaptation of the measure and evaluation of its cross-cultural applicability are crucial [17].

Previous studies on tool validation in other languages have shown satisfactory reliability and validity of the PEDT [5]. Our results have also shown good correlation and internal consistency between the English version and PEDT-U. The internal consistency demonstrated by Cronbach's alpha (α = 0.939) was acceptable. Similarly, for the Iranian version of PEDT, the test-retest correlation coefficients for each item in both the patient and control groups were higher than 0.81, and the correlation coefficients for the total score were 0.92 and 0.94 in the patient and control groups, respectively [17].

We have found that PEDT-U is an effective tool to diagnose PE. It correctly diagnosed PE with a sensitivity of 0.736 and correctly identified individuals without PE with a specificity of 0.988. The sensitivity and specificity analyses of the original PEDT used cutoff scores of ≤8 for no PE and ≥9 for PE [5]. However, further studies are required to address the needs of the illiterate population in Pakistan.

The most important finding in the study was the ease in completing the Urdu version as compared to the English version. This was demonstrated by the time taken to fill each version. The time taken to complete the PEDT-U was significantly shorter. This means that the local bilingual population, fluent in English and Urdu, was more at ease with the Urdu version of their condition. Most participants reported in subsequent interviews that their responses in the Urdu version were more in line with their feelings than those in the English version.

## Conclusions

The Urdu version of PEDT (PEDT-U) has shown good correlation and internal consistency with the original English version of PEDT. It takes less time, and patients feel more comfortable with it. Hence, it can be used to establish the diagnosis and find out the severity of PE in the Pakistani population. Hence, it can be used among the literate population of Pakistan. However, further studies are required to address the needs of the illiterate population in Pakistan.
